# Genotoxic Effects of Cylindrospermopsin, Microcystin-LR and Their Binary Mixture in Human Hepatocellular Carcinoma (HepG2) Cell Line

**DOI:** 10.3390/toxins12120778

**Published:** 2020-12-08

**Authors:** Leticia Díez-Quijada, Klara Hercog, Martina Štampar, Metka Filipič, Ana M. Cameán, Ángeles Jos, Bojana Žegura

**Affiliations:** 1 Area of Toxicology, Faculty of Pharmacy, Universidad de Sevilla, Profesor García González No. 2, 41012 Seville, Spain; ldiezquijada@us.es (L.D.-Q.); camean@us.es (A.M.C.); 2 Department of Genetic Toxicology and Cancer Biology, National Institute of Biology, Večna pot 111, 1000 Ljubljana, Slovenia; klara.hercog@nib.si (K.H.); martina.stampar@nib.si (M.Š.); metka.filipic@nib.si (M.F.); bojana.zegura@nib.si (B.Ž.); 3 Jozef Stefan International Postgraduate School, Jamova 39, 1000 Ljubljana, Slovenia

**Keywords:** DNA double-strand breaks, cell-cycle, mRNA expression, microcystin-LR, cylindrospermopsin, mixture, HepG2 cells

## Abstract

Simultaneous occurrence of cylindrospermopsin (CYN) and microcystin-LR (MCLR) has been reported in the aquatic environment and thus human exposure to such mixtures is possible. As data on the combined effects of CYN/MCLR are scarce, we aimed to investigate the adverse effects related to genotoxic activities induced by CYN (0.125, 0.25 and 0.5 µg/mL) and MCLR (1 µg/mL) as single compounds and their combinations in HepG2 cells after 24 and 72 h exposure. CYN and CYN/MCLR induced DNA double-strand breaks after 72 h exposure, while cell cycle analysis revealed that CYN and CYN/MCLR arrested HepG2 cells in G0/G1 phase. Moreover, CYN and the combination with MCLR upregulated *CYP1A1* and target genes involved in DNA-damage response (*CDKN1A*, *GADD45A*). Altogether, the results showed that after 72 h exposure genotoxic activity of CYN/MCLR mixture was comparable to the one of pure CYN. On the contrary, MCLR (1 µg/mL) had no effect on the viability of cells and had no influence on cell division. It did not induce DNA damage and did not deregulate studied genes after prolonged exposure. The outcomes of the study confirm the importance of investigating the combined effects of several toxins as the effects can differ from those induced by single compounds.

## 1. Introduction

Cyanobacteria are organisms ubiquitously present in different environments ranging from marine and freshwaters to hydrothermal vents, and from Antarctic lakes to desert rocks [1]. Nowadays blooming of cyanobacteria is increasing all over the world due to anthropogenic activities and climate changes [2]. Cyanobacteria produce biologically active substances including cyanotoxins ranging from hepatotoxins, cytotoxins, neurotoxins, dermatotoxins, and irritant compounds with diverse structural and physicochemical properties [3,4,5].

**Figure 1:**
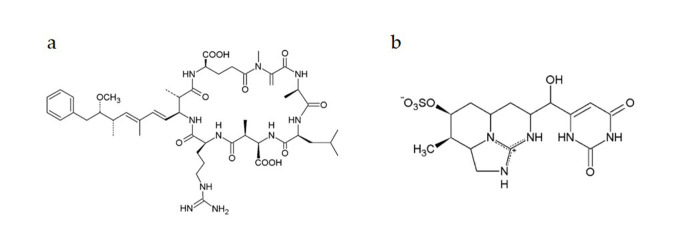
Chemical structure of MCLR (**a**) and CYN (**b**).

One of the most widely known groups of hepatotoxic cyanotoxins are microcystins (MCs) that are produced by different genera of cyanobacteria such as *Microcystis, Planktothrix* and *Dolichospermum* among others [6]. They are cyclic heptapeptides (Figure 1a), and to date, at least 279 MCs variants have been recognized [7], with microcystin-LR (MCLR) congener as the most studied due to its toxicity and abundance in comparison to other MC-congeners [2,8,9]. Mainly it targets the liver, due to its active transport via hepatic organic anion transport polypeptides (OATP) [10]. The inhibition of specific protein serine/threonine phosphatases, types 1 and 2A (PP1 and PP2A) is the most largely investigated mechanism of action of MCLR [2,11]. Nonetheless, the mechanisms involved in MCLR toxicity are not completely elucidated, and in addition to PP1/PP2A inhibition also induction of oxidative stress [2,12,13] and apoptosis [14] have been described. It has been proved that MCLR can induce genotoxic effects [4,11], and can act as a tumor promoter [15]. Consequently, this can lead to cancer development especially after long-term exposure to low concentrations of MCLR [16,17]. Due to MCLR known adverse genotoxic and tumor-promoting potential, the International Agency for Research on Cancer (IARC) classified the toxin within the group 2B as “a possible human carcinogen” [18]. 

Cylindrospermopsin (CYN), another very important cyanotoxin, has been recently considered as an emerging toxin. It is produced by many cyanobacterial genera, with *Cylindrospermopsis raciborskii* and *Chrysosporum ovalisporum* being its main producers [19]. It is a tricyclic alkaloid with a guanidine group linked to a hydroxymethyluracil group [20], and its zwitterionic character makes it a highly soluble compound in water [21] (Figure 1b). CYN mainly targets the liver, although other organs may be affected as well suggesting its cytotoxic properties [22,23,24]. Among the CYN toxic mechanisms, the irreversible inhibition of protein [25,26] and glutathione (GSH) synthesis have been described [26,27,28,29] and revised by Pichardo et al. [30]. Similarly to MCs, CYN mechanisms of action are not fully elucidated, and many studies reported that it has to be metabolized by cytochrome P450 to show cytotoxic and genotoxic activities, which points out that CYN is pro-genotoxic [4,28,29,31,32,33,34,35,36]. Moreover, CYN has been reported to cause DNA single (SSB) and double (DSB) strand breaks and induces genomic instability [29,30,31,32,34,35,37,38,39,40,41]. Nevertheless, additional studies are needed to completely understand its genotoxic properties. 

In the environment, many cyanobacterial species can co-exist producing various toxins at the same time as it has been reported for MCLR and CYN [1,42,43,44,45,46], meaning that organisms living in such areas can be simultaneously exposed to complex mixtures of toxins. Due to distinct mechanisms of action of toxins present in such complex mixtures, synergistic, potentiating or antagonistic interactions can occur. Thus, it is essential to study the combined adverse effects of cyanotoxins, as these can vary from those induced by single compounds. In order to perform an appropriate risk assessment and hazard identification, the European Food Safety Authority (EFSA) [1] recommended to evaluate possible adverse effects of mixtures. In this regard, a previous study suggested that CYN had a prominent role in the genotoxicity of CYN/MCLR combination by the comet assay, the micronucleus assay and target gene expression on HepG2 cells [34], but further research was required.

Therefore, we aimed to evaluate cytotoxic and genotoxic activity of MCLR, CYN, and their binary mixture using the metabolically active human hepatocellular carcinoma (HepG2) cell line as experimental model. Genotoxic activity was evaluated by detecting the H2AX phosphorylation that reflects an early event to a genotoxic insult with subsequent formation of DNA DSB. This is very important as genotoxic compounds damage DNA, which can be manifested in the next cell generation, leading to mutations or other genetic damages. Further, flow cytometry was used to evaluate the impact of MCLR, CYN and their binary mixture on the cell cycle for the first time, which can add information for the explanation of possible mechanisms of action. Previously, these two assays have been applied only regarding CYN exposure, but the effects of the combination has been not investigated so far. The underlying mechanisms involved in toxic activities of studied toxins were also evaluated by analyzing the changes in the mRNA expression of important targeted genes implicated in the metabolism of xenobiotic substances (*CYP1A1* and *UGT1A1*) and DNA damage response (*TP53, MDM2, CDKN1A, GADD45A* and *JUNB*) after a prolonged exposure time (72 h), not previously considered. To study the delayed effects and to check whether the harmful effects persist in cell population is also relevant to understand the (geno)toxic effects.

## 2. Results

### 2.1. Viability of HepG2 Cells Exposed to CYN, MCLR and Their Mixtures

**Figure 2:**
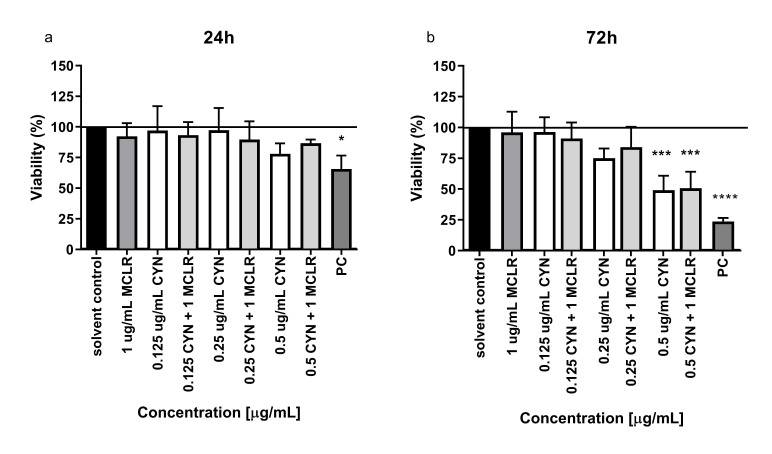
The influence of graded CYN concentrations (0.125, 0.25 and 0.5 µg/mL, [white]), MCLR (1 µg/mL [grey]) and CYN/MCLR [light grey] mixtures on HepG2 cell viability. Viability was detected with the MTS assay after 24 h (**a**) and 72 h (**b**) of exposure. The results are presented as mean values ±SD of three independent experiments (* *p* < 0.05; *** *p* < 0.001; **** *p* < 0.0001). Positive control (PC) was etoposide (ET) at 30 µg/mL.

Cytotoxic effects of CYN and MCLR and their mixtures were assessed after 24 and 72 h of exposure on HepG2 cells. After 24 h, no significant influence on cell viability was determined after CYN, MCLR nor CYN/MC-LR mixtures (Figure 2a). In contrast, after prolonged exposure of 72 h to CYN (0.5 µg/mL) alone and in combination with MCLR (1 µg/mL) a significant decrease in cell viability of up to 50% compared to solvent control was determined. No significant differences in cell viability at any exposure time were determined between non-treated control and solvent control cells (data not shown).

### 2.2. The Formation of DNA Double Strand Breaks (DSBs) Induced by CYN, MCLR and Their Binary Mixture

**Figure 3:**
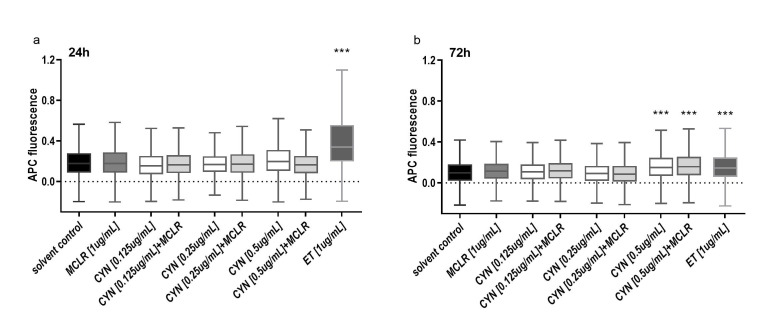
The formation of DNA DSBs in HepG2 cells after the exposure to CYN (0.125, 0.25 and 0.5 µg/mL, [white]), MCLR (1 µg/mL [grey]), and CYN/MCLR mixtures [light grey]. DSBs were determined by flow cytometry by detection of H2AX phosphorylation after 24 h (**a**) and 72 h (**b**) of exposure. The results are presented in quantile box plots. Three independent experiments were performed with 10^4^ events recorded in each sample per experiment (*** *p* < 0.001). Positive control was Etoposide (ET, 1 µg/mL).

The induction of DNA DSBs by CYN, MCLR and their binary mixtures was studied after 24 and 72 h exposure by detection of γH2AX positive cells using flow cytometry. After 24 h no significant elevation in DSBs formation was determined at any concentration applied (Figure 3a), while after 72 h CYN at 0.5 µg/mL and the CYN/MCLR mixture (0.5 µg/mL/1 µg/mL) (Figure 3b) induced a statistically significant increase in the formation of DSBs. MCLR at 1 µg/mL did not increase the formation of DSBs at any exposure time.

### 2.3. The Influence of CYN, MCLR and Their Binary Mixture on the Cell-Cycle Progression

**Figure 4:**
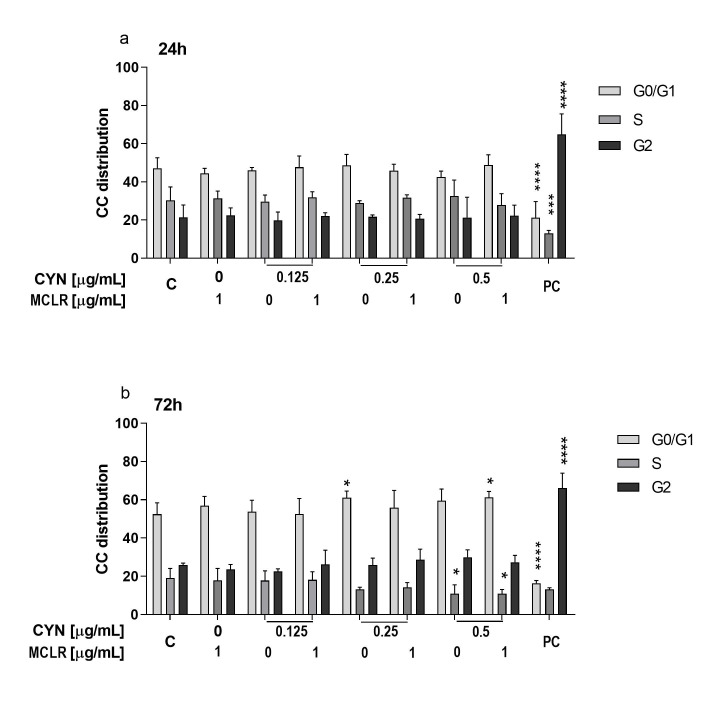
The influence of CYN (0.125, 0.25 and 0.5 µg/mL), MCLR (1 µg/mL) and CYN/MCLR mixtures on the distribution of HepG2 cells across the cell cycle phases. Cell-cycle was analyzed by flow-cytometry after 24 h (**a**) and 72 h (**b**) of exposure. Three independent experiments were performed with 10^4^ events recorded in each sample per experiment (* *p* < 0.05; *** *p* < 0.001; **** *p* < 0.0001). Positive control (PC): 1 µg/mL Etoposide.

The influence of CYN, MCLR and their binary mixtures on HepG2 cell-cycle progression after 24 and 72 h exposure was determined by flow cytometry. After 24 h, CYN (0.125–0.5 μg/mL), MCLR (1 μg/mL) and the mixtures did not significantly influence the cell-cycle (Figure 4a). After 72 h, CYN alone (0.25 μg/mL) and CYN (0.5 μg/mL) in combination with MCLR (1 μg/mL), arrested cells in G0/G1 phase, while CYN at 0.5 μg/mL showed the same trend, but did not statistically significantly differ from the solvent control. We observed a concomitant decrease of the number of cells in the S phase after the exposure to CYN (0.5 µg/mL) alone and the combination with MCLR (1 µg/mL) (Figure 4b).

The key decisions on DNA replication and finalization of the cell division are done in the G1 phase of the cell-cycle. As DNA damage activates p53 protein that controls the transcription of genes implicated in the arrest of the cell cycle in G1/S phase, including the cyclin-dependent kinase inhibitor 1 (P21) [47], present results suggest the induction of genotoxic stress by CYN and the combination with MCLR. The PC (ET 1 µg/mL) had a significant effect on cell distribution throughout the cell cycle after both exposure times.

### 2.4. The Effects of CYN, MCLR and Their Mixtures on the mRNA Expression

The mRNA expression of studied genes implicated in the metabolism, response to DNA damage and immediate-early response/signaling was analyzed after 72 h of exposure to CYN (0.5 µg/mL), MCLR (1 µg/mL) and their combination by qPCR, using as positive control B(a)P 30µM. The results are present in Table 1 and are shown as relative expression of the corresponding gene.

The mRNA expression of *CYP1A1* encoding an enzyme belonging to the cytochrome P450 family, which is involved in the metabolism of xenobiotics and drugs (phase I), was up-regulated by 2.59- and 2.53-fold in HepG2 cells exposed to CYN (0.5 µg/mL) and the CYN/MCLR combination, respectively. The mRNA level of *UGT1A1*, a member of phase II of xenobiotic metabolism, was not affected after any of the exposures applied in the study (pure cyanotoxins and their combinations).

Further, we analyzed the expression of *JUNB*, a gene that is implicated in immediate-early response/signaling, and the results showed that pure cyanotoxins and their combination did not deregulate the expression of *JUNB* at any exposure.

After 72 h, CYN and its combination with MCLR significantly down-regulated the expression of *TP53* for -1.31-fold and -1.33-fold, respectively. *MDM2* was not deregulated by CYN, MCLR nor the combination of both cyanotoxins. DNA damage responsive genes *CDKN1A* (2.44-fold and 2.47-fold, respectively) and *GADD45A* (3.16-fold and 3.06-fold, respectively) were significantly up-regulated by CYN and CYN/MCLR combination, respectively.

## 3. Discussion

Cyanobacteria produce many biologically active secondary metabolites that can induce adverse health effects and can negatively influence water quality and the ecosystem [4,48]. Despite the fact that humans and animals are in their everyday life exposed to complex cyanobacterial mixtures, the studies describing the combined effects of these toxins are scarce. Among the most studied cyanotoxins are MCLR and CYN that frequently occur in cyanobacterial blooms simultaneously [1,42,43,44,45,46]. Both cyanotoxins are known to induce cytotoxic and genotoxic effects, but their mechanisms of action differ [2,4,11,30]. Although the effects of cyanotoxins combination has been previously investigated by our research group [34], we aimed to assess this time long-exposure responses to observe the induction of delayed effects or to study if the harmful effects persist in cell population.

In our study, cytotoxic and genotoxic activities of MCLR and CYN and their combinations were studied in HepG2 cells. This experimental model was selected based in our previous studies [34] and its reported sensitivity to cyanotoxins as it has been demonstrated in the scientific literature. The obtained results are in agreement with published data describing a decrease of HepG2 cell viability upon exposure to pure CYN and its combination with MCLR [34,49]. Similar CYN/MCLR combined cytotoxic effects were observed also in human neuroblastoma SH-SY5Y cell line [50].

MCLR is known to induce DNA strand breaks mostly single-strand breaks that are formed transiently as a result of oxidative damage repair process [12]. CYN on the other hand is pro-genotoxic and needs metabolic activation for its genotoxic activity. In addition to DNA strand breaks, it induces chromosomal damage [29,31,32,34,38,39], while MCLR does not influence genomic instability of HepG2 cells [34]. CYN is considered a clastogen, thus it is expected to form DNA DSBs. DSBs are an extremely damaging form of DNA damage as they can cause mutations and chromosomal rearrangements [51]. The formation of DSB is followed by phosphorylation of the histone H2AX [52]. Thus, the detection of γ-H2AX positive cells by flow cytometry can be considered as a DNA damage biomarker [53]. Previously it has been described that CYN after 72 h exposure increases the formation of DNA DSBs in HepG2 cells [39,41], while no data for MCLR exist. Our results confirmed an increase in γ-H2AX positive cells that corresponds to DNA DSBs formed after 72 h exposure to CYN, while no increase in γ-H2AX formation was detected after 24 h. On the other hand, no induction of DNA DSB by MCLR at any of the exposure times was detected. This is in line with the previously reported MCLR induced lesions. Namely, the toxin induces DNA single-strand breaks that are repaired after prolonged exposures [12] and it does not induce micronuclei in HepG2 cells [34]. It is known that the formation of micronuclei correlates with DNA double-strand break formation [54]. When comparing the effects induced by pure CYN and the combination with MCLR, no significant differences were determined, suggesting that induced DNA DSB were the consequence of CYN activity. Similarly, it has been reported for micronuclei formation where CYN induced genomic instability in HepG2 cells to a similar extent as the combination with MCLR [34]. Moreover, CYN/MCLR mixtures have been shown to increase the frequency of micronuclei in cultured mouse lymphoma cells L5178YTk^±^ compared to control [55].

Cells respond to DNA damage by triggering a complex network of check-point kinases to delay their cell-cycle progression and repair the lesions [56,57]. In the cell cycle G1 phase, key decisions on DNA replication and finalization of the cell division are done. Upon genotoxic stress, P53 protein is activated and this leads to the regulation of transcription of those genes that are implicated in the arrest of cell cycle in G1/S phase, including the cyclin-dependent kinase inhibitor 1 (P21) [47]. Therefore, in our study, we investigated the impact of CYN, MCLR and CYN/MCLR combinations on the cell cycle progression. After 24 h, no significant changes in the cell cycle were determined, while after 72 h CYN and CYN/MCLR combination at the highest concentration tested affected the cell cycle. In comparison to solvent control, we determined increased frequency of cells in G0/G1 phase in CYN (0.25 µg/mL) and CYN/MCLR (at 0.5 µg/mL/1 µg/mL) exposed cells together with reduced number of cells in S phase. Similar to our results, CYN arrested the cells in G0/G1 phase in HepG2 spheroids [36] and human T-lymphocytes [58]. Contrary to this, Štraser et al. [39] described that CYN (0.5 μg/mL) decreased the frequency of HepG2 cells in G0/G1 phase after 72 h and at the same time arrested cells in S phase of the cell cycle. DNA synthesis arrest in the S-phase is specifically associated to DNA DSBs via the p53-independent ATM pathway [59].

Here for the first time, the influence of MCLR on the cell cycle distribution was studied and the results showed that after 24 and 72 h MCLR had no influence on the distribution of cells in the cell cycle. This is in line with the results describing that MCLR at prolonged exposures does not induce DNA SSB nor DSB and does not affect genomic instability of HepG2 cells. Previously, Lankoff et al. [60] described that MCLR at high concentrations (>25 µM) in non-target Chinese hamster ovarian (CHO-K1) cells caused the accumulation of cells in mitosis and thereby increased mitotic index (MI) was reported after 18 h of exposure. In addition, concentration dependent elevation in the number of abnormal anaphases and polyploid metaphases together with elevated cell death by apoptosis and necrosis has been described. The authors explained these effects as a consequence of MCLR inhibition of protein phosphatases PP1A and PP2A that are implicated in cell-cycle progression with an important role in mitosis. On the contrary, Liu et al. [61] did not observe disorders of cell-cycle distribution after the exposure of liver cell line HL7702 to MCLR (10 µM) for 36/48 h.

We further studied the mechanisms implicated in the toxicity of CYN and MCLR as single compounds, and their combination by analyzing their influence on the mRNA expression of *CYP1A1* and *UGT1A1* encoding the metabolic enzymes from phase I and II, respectively. The cytochrome P-450 (CYP450) enzymes are membrane-bound hemoproteins, which have an important role in the activation/detoxification of xenobiotic compounds, homeostasis, and cellular metabolism. Activation and/or inhibition of CYPs is a primary mechanism for metabolism-based chemical-chemical interactions. CYP450 enzymes are transcriptionally activated by endogenous substrates and xenobiotic compounds via receptor-dependent mechanisms. As a consequence, CYP-mediated biotransformation leads to metabolic activation of chemicals and environmental compounds to reactive products with carcinogenic activity [62]. It is generally known that CYN is a pro-genotoxic and has to be metabolically activated by CYP450 enzymes to induce genotoxic effects, which was confirmed in several in vitro [29,31,32,34,36,38,39,41,63,64] and in vivo [26] models. We investigated the influence of cyanotoxins on the gene expression of *CYP1A1*, which is one of the key P450 enzymes that in humans participates in the metabolic transformation of most xenobiotics and is implicated in the bio-activation of many pro-carcinogenic compounds to reactive metabolites [65]. Previously, Hercog et al. [34] showed that in HepG2 cells, CYN (0.5 μg/mL), MCLR (1 μg/mL) and the combination CYN/MCLR (0.5 μg/mL/1 μg/mL) did not deregulate *CYP1A1* after short exposure time (4 h), while after 24 h exposure CYN upregulated the expression of *CYP1A1* to a similar extent as the combination of both toxins suggesting that the up-regulation of *CYP1A1* occurred due to CYN activity. MCLR alone, on the contrary, did not elevated the level of mRNA in HepG2 cells after 24 h [34]. In our study, we prolonged the treatment time to 72 h. The results showed that CYN and the combination CYN/MCLR increased the *CYP1A1* mRNA level to a similar extent; hence, the changes induced by CYN/MCLR combination could be attributed to CYN, especially as MCLR had no effect on the expression of this gene. This is in agreement with the current knowledge on MCLR metabolic pathway, as glutathione conjugation that is catalyzed by glutathione-S-transferases or occurs spontaneously, is the main accepted detoxification step of MCs [2] with no published reports on bioactivation step confirming the assumptions that MCLR is not metabolically transformed. The expression of *UGT1A1* gene, that is encoding one of the most important enzymes of phase II, UDP glucuronosyltransferase 1 family polypeptide A1, involved in the detoxification of xenobiotic compounds, was not deregulated by CYN, MCLR nor the combination of these two cyanotoxins after prolonged exposure. Similar observations were reported also after treatment of HepG2 cells for 4 and 24 h [34]. In opposite, CYN up-regulated *UGT1A1* expression in HepG2 spheroids [36].

The P53, a tumor suppressor protein, plays a key role in the pathways related to DNA damage response including the regulation of cell cycle progression, differentiation, repair of DNA damage, apoptosis, and senescence [66]. In response to DNA damage, the p53 protein is mostly activated via the phosphorylation by DNA damage responsive kinases and, to a minor part, by the up-regulation of gene expression [67]. Seventy-two hour treatment of HepG2 cells with CYN, MCLR or CYN/MCLR mixture did not importantly deregulate the expression of *TP53*, which corroborates previously published studies describing no influence of CYN [32,34], MCLR [34] or the combination [34] on the mRNA level of *TP53*. At most, we noticed slight down-regulation of *TP53* upon CYN and CYN/MCLR exposure, which was for CYN recently described also by Hercog et al. [41]. The p53 network is activated by DNA damage, which induces the transcription of genes that are regulated by p53, including *MDM2*, *GADD45A* and *CDKN1A*. These genes are recognized as important molecular biomarker genes of genotoxic and carcinogenic stress [68,69,70]. *MDM2* that encodes the enzyme implicated in the degradation of p53 and thus, stimulates the progression of cell cycle and increases tumorigenic potential [71], was not deregulated in HepG2 cells upon 72 h exposure to CYN, MCLR or their combination. Similar observations were reported by Hercog et al. [34] after 4 and 24 h treatment of HepG2 cells to CYN, MCLR and the combination of both toxins, while Štraser et al. [32] described slight 1.6-fold up-regulation of *MDM2* in HepG2 cells exposed to CYN for 24 h [34]. We further studied the impact of cyanotoxins on the expression of genes implicated in the response to DNA damage, namely *CDKN1A* and *GADD45A.* P21, a cyclin-dependent kinase inhibitor (coded by *CDKN1A*), induces cell cycle arrest (prevents G1-S transition) in response to various stimuli such as DNA damage, and thus inhibits DNA replication in addition to regulating essential processes, such as apoptosis and transcription [72,73]. Moreover, the expression of *GADD45* gene can be induced by diverse pathways like genotoxic stress and multiple environmental and physiological stresses [74]. Besides, the protein is significantly involved in the control of the cell-cycle at G2-M checkpoint, induction of DNA repair and apoptosis [75]. Both genes were significantly upregulated upon 72 h exposure to CYN and CYN/MCLR to a very similar extent, while the expression of these genes was not deregulated by pure MCLR. These results corroborate with the observation described by Hercog et al. [34]. The authors noticed up-regulation of *CDKN1A* and *GADD45A* by CYN and its combination with MCLR after 24 h of exposure at the same concentrations as used in our study, while after short exposure of 4 h no deregulation of these two genes was noticed. Based on the results of our study, it could be concluded that CYN and CYN/MCLR mixture induced similar time-dependent up-regulation of genes implicated in DNA damage response, confirming the induction of DNA lesions and their subsequent accumulation in HepG2 cells resulting from CYN exposure. On the other hand, MCLR alone had no influence on the expression of genes that respond to DNA damage, *CDKN1A* and *GADD45A*. This is in line with results describing that in HepG2 cells after prolonged exposures MCLR does not induce DNA single [34] nor double-strand breaks (present study) nor micronuclei [34].

From the genes belonging to the group of immediate-early response/signaling, we evaluated the expression of *JUNB* gene, which exerts a dual action on the cell-cycle. It is well known for its role as an inhibitor of cell proliferation, an inducer of senescence and a tumor suppressor; however, it is also known for its cell-division-promoting activity [76]. *JUNB* was reported to be upregulated in HepG2 cells exposed to CYN and CYN/MCLR mixture for 24 h [34]. However, the results of our study showed that after prolonged exposure of 72 h none or the studied cyanotoxins nor their combination deregulated *JUNB*.

## 4. Conclusions

CYN and MCLR are two ubiquitously found cyanotoxins, which in the aquatic environment occur simultaneously meaning that humans and animals can be in their real life exposed to their combinations. However, the effects of their mixtures have been not yet widely investigated. Both toxins induce DNA damage, but their mechanisms of action differ. In the present study, cyto/genotoxic effects of CYN and MCLR and their co-exposure in metabolically competent HepG2 cells were investigated. Viability of cells was significantly affected by pure CYN and its combination with MCLR after prolonged exposure. CYN and CYN/MCLR combination increased the frequency of DNA DSBs, being CYN/MCLR evaluated for the first time. In general, no significant differences were determined between CYN and the CYN/MCLR treated cells, indicating that observed effects were due to CYN activity. The gene expression analysis after prolonged exposure time (72 h) again indicated that CYN/MCLR mixture showed very similar pattern as CYN alone. Strong up-regulation of metabolic gene *CYP1A1* that is upregulated upon exposure to indirect-acting xenobiotic compounds again showed that metabolism plays a key role in the activation of CYN and thus its cyto/genotoxic potential, but not in the case of MCLR. The up-regulation of genes implicated in DNA damage responsive, *CDKN1A*, and *GADD45A* in the presence of CYN, confirmed its genotoxic activity. As the mixture of CYN/MCLR showed a very similar pattern in the gene expression as well as induction of DNA damage when compared to pure CYN and no additive/synergistic effects could be noticed, it can be concluded that the observed changes are due to the effects of CYN alone, which presents by far greater risk for human exposure than MCLR.

## 5. Materials and Methods

### 5.1. Chemicals

Microcystin-LR (99% purity) and Cylindrospermopsin (95% purity) were purchased from Alexis Corporation (Lausen, Switzerland). Stock solutions of CYN (0.5 µg/mL) and MCLR (1 µg/mL) were prepared in 50% methanol and pure ethanol (99.8%), respectively. Etoposide (ET) (Santa Cruz Biotechnology, St. Cruz, CA, USA) and benzo(a)pyrene (B(a)P) (Sigma, St. Louis, MO, USA) were prepared in dimethylsulphoxide (DMSO) at 25 mg/mL and 9.9 mM, respectively. MEM, methanol ethanol, DMSO, trypsin and PMS (phenazine methosulfate) were provided from Sigma, St. Louis, MO, USA. The MTS was from Promega (Madison, WI, USA). L-glutamine, phosphate buffered saline (PBS), penicillin/streptomycin, and foetal bovine serum (FBS), were from PAA Laboratories (Dartmouth, NH, USA). TRIzol^®^ and Hoechst 33,342 were provided from Invitrogen (Paisley, Scotland, UK). REA Control (I)-APC human and anti-H2AX pS139-APC human were bought from MACS Miltenyi Biotec (Bergisch Gladbach, Germany). The cDNA High Capacity Archive Kit, Taqman Gene Expression Assays, and Taqman Universal PCR Master Mix were purchased from Applied Biosystems (Waltham, MA, USA).

### 5.2. Cell Culture

Human hepatocellular carcinoma (HepG2) cell line was bought from the ATCC—HB-8065™ (Manassas, VA, USA). This cell line has been selected as an in vitro experimental model as it retained inducibility and activities of several phase I and phase II xenobiotic metabolizing enzymes, which is important for detection of different classes of indirect acting genotoxic agents [77]. Moreover, HepG2 cells express wild-type tumor suppressor TP53 [78], which makes them a suitable model for studying P53 regulated response to DNA damage at the level of gene transcription and translation [79] The cell line has been extensively utilized as in vitro alternative model to primary human hepatocytes for drug metabolism and hepatotoxicity studies [80]. Cells were cultured in MEM medium supplemented with 1% NEAA, 2 mM L-glutamine, 10% FBS, 100 IU/mL penicillin/streptomycin, 1 mM sodium pyruvate, and 2.2 g/L NaHCO_3_ at 37 °C and 5% CO_2_. Cells were checked for mycoplasma by MycoAlert^TM^ kit from Lonza (Walkersville, MD, USA).

### 5.3. Concentrations of CYN, MCLR and Their Combinations for Determining Cytotoxicity, Genotoxicity and mRNA Expression

Before the treatment, cells were seeded into 96-wells Nunc plates (TermoFisher Scientific, Waltham, MA, USA) (8000 and 4000 cells/well for 24 and 72 h, respectively) to assess cytotoxicity with MTS assay and 25 cm^2^ culture flask (Corning Costar Corporation) (800,000 and 400,000 cells/plate for 24 and 72 h, respectively) for flow cytometry and gene expression analyses. The cells were left for 24 h at 37 °C in 5% CO_2_. Subsequently, they were treated with CYN (0.125, 0.25 and 0.5 µg/mL) and MCLR (1 µg/mL) as single compounds and CYN/MCLR combinations for 24 and 72 h when evaluating the influence of toxins on the induction of DNA DSBs and cell cycle. The gene expression analysis was evaluated only after 72 h exposure of cells to CYN (0.5 µg/mL), MC-LR (1 µg/mL) and CYN/MCLR combination. Etoposide (30 and 1 µg/mL) was applied as a positive control for MTS and flow cytometry analyses, respectively, while B(a)P (30 µg/mL) was used for gene expression analyses. CYN and MCLR concentrations were selected based in our previous research [34] and the environmental levels [2]. Growth medium (negative control) and corresponding solvent control (medium supplemented with both 0.1% ethanol and 0.05% methanol) were included in all experiments. No statistically significant difference was determined between negative and solvent control; therefore, only the results for solvent control are presented.

### 5.4. Cytotoxicity Assay-MTS Test

Viability of HepG2 cells was determined after 24 and 72 h of exposure to pure cyanotoxins and their combinations with the MTS tetrazolium reduction assay in accordance with the manufacturer’s protocol. At the end of the incubation, 20% of fresh MTS: PMS mixture (20:1) was added to treated cells and incubated for 3 h. The viability of cells was evaluated by measuring the optical density (490 nm) using a spectrofluorimeter (Sinergy Mx, BioTek, Winooski, VT, USA), which correlates to the amount of viable cells that are able to reduce tetrazolium to formazan. Three independent experiments were done, for each treatment time in five replicates per treatment point. Statistical analysis among treatments and solvent control was performed by One-way analysis of variance (Dunnett’s Multiple Comparison Test) with GraphPad Prism 6 (GraphPad Software, La Jolla, CA, USA) (* *p* < 0.05).

### 5.5. Influence of Cyanotoxins on DNA Double-Strand Break Induction and Cell Cycle Determined by Flow Cytometry

After the treatment, the adherent and floating cells were joint by trypsinization. Subsequently, the cells were washed twice with 2 mL cold PBS, centrifuged (1000 rpm, 4 °C, 5 min) and resuspended in cold PBS (0.5 mL). For fixation the ethanol (1.5 mL of 96% ethanol) was added dropwise during vortexing and the cells were left at 4 °C for two hours and stored until analysis at −20 °C. Before labelling with antibodies, fixed cells were left at room temperature for 10 min and were then centrifuged (1300 rpm, 10 min), washed two times with 3 mL ice-cold PBS and centrifuged again (1300 rpm, 5 min).

Simultaneous detection of DNA DSBs and analysis of cell cycle was performed by flow cytometry as follows: after washing, the cells were resuspended in a mixture of 0.1 mL BSA (0.15 M) and Anti-γH2AX pS139 (proportion 3:50) antibody in accordance with the manufacturer’s protocol. Cells were subsequently incubated (30 min) at room temperature in the darkness. γH2AX-labelled cells were then washed once with 1 mL 1% BSA and stained with Hoechst 33,258 dye that was diluted in 0.1% Triton X-100 in the ration 1:500 (30 min at room temperature in the darkness). Stained cells were then washed once with 1 mL 1% BSA and resuspended in 1 × PBS (0.2 mL). To exclude unspecific binding, the rea-APC control (Miltenyi Biotec, Germany) was used. Flow cytometric analyses were performed on a MACSQuant Analyzer 10 (Miltenyi Biotech, Germany). APC intensity, that corresponded to DSBs, was identified in R1 (655–730 nm) channel and Hoechst fluorescence related to DNA content for cell cycle analyses was determined in V1 (450/500 nm) channel. Three independent experiments were performed, where 10.000 events per sample in each experiment were recorded. For further analysis of double-strand breaks, the raw data obtained from flow cytometer were exported to GraphPad Prism 6 to make the box and whiskers plots and the statistical significance in the change of APC fluorescence was calculated with the linear mixed-effects model (nlme) by REML in the R program [81] (*** *p* < 0.001). Moreover, the % of cells in each phase of the cell cycle (G0/G1, S, and G2/M) was evaluated by FlowJo Software using Dean jet fox model (Single Cell Analysis Software v10, FlowJo, LLC). The statistical significance between solvent control and treated groups was analyzed by Kruskal-Wallis test followed by Dunn’s multiple comparison test using Graph-Pad Prism 6 software (Graph-PadSoftware Inc., La Jolla, CA, USA) (* *p* < 0.05).

### 5.6. Gene Expression Analysis by Quantitative Real-Time PCR (qPCR)

The expression of studied genes was determined by qPCR after 72 h treatment of HepG2 cells to cyanotoxins and their combination as explained in Section 5.3. Afterwards, total RNA was isolated with TRizol^®^ (Gibco BRL, Paisley, Scotland) reagent in accordance with the manufacturer’s protocol for each cyanotoxin, their mixture and controls. The purity and concentration of isolated mRNA were evaluated using NanoDrop 1000 Spectrophotometer (Thermo Fisher Scientific, Waltham, MA, USA). The mRNA (1 µg per sample) was reverse transcribed to cDNA using cDNA High Capacity Archive Kit. Quantification of studied genes was carried out by qPCR method using TaqMan Universal PCR Master Mix and the following Taqman Gene Expression Assays were applied: *CYP1A1* (cytochromeP450, family 1, subfamily A, polypeptide 1) Hs01054797_g1 and *UGT1A1* (UDP glucuronosyltransferase1 family, polypeptide A1) Hs02511055_s1 for assessment of metabolic activity; *JUNB* (jun B proto-oncogene) Hs00357891_s1, for immediate/early response markers; *TP53* (tumor protein P53), Hs01034249_m1, *GADD45A* (‘growth arrest and DNA damage-inducible gene, alpha’), Hs00169255_m1, *CDKN1A* (Cyclin-Dependent Kinase Inhibitor 1A) Hs00355782_m1, and *MDM2* (Mdm2, ‘MDM2 oncogene, E3 ubiquitin-protein ligase) Hs00234753_m1 for assessment of DNA-damage response. As an internal control in all experiments GADPH was applied (Human Endogenous Controls, 4310884E, Applied Biosystems, USA). The following conditions for the qPCR were used: 50 °C for 2 min, 95 °C for 10 min and 45 cycles of 95 °C for 15 s and 60 °C for 1 min. To eliminate the inhibition effect, a serial of 10-fold dilutions of each target gene was analyzed in the control sample. The qPCR experiments were run on VIA Real-Time PCR System machine (The Applied Biosystems™). Relative quantification in accordance to the solvent control with an open web program quantGenius was used for data analysis [82]. Three independent experiments were performed. Significance differences between solvent control and treated groups were evaluated by One-way analysis of variance (Dunnett’s Multiple Comparison Test). (* *p* < 0.05, ** *p* < 0.01, *** *p* < 0.001 and **** *p* < 0.0001). A positive response in respect to the solvent control group was considered when differences ≥1.5-fold (up-regulation) and ≤0.66-fold (down-regulation) were observed.

## Figures and Tables

**Table 1:** The effects of CYN (0.5 µg/mL), MCLR (1 µg/mL) and CYN/MCLR combination in HepG2 cells after 72 h of exposure on the mRNA level of studied genes implicated in the metabolism, DNA damage response and immediate-early response/signaling. The results are shown as relative expression of mRNA and are normalized to the solvent control. B(a)P 30 µM was used as positive control. Mean values (±SD) of three independent experiments are calculated (* *p* < 0.05, ** *p* < 0.01, *** *p* < 0.001, **** *p* < 0.0001; One-way analysis of variance and Dunnett’s Multiple Comparison Test. Bold values present up or down-regulation of genes with the threshold of 1.5-fold (≥1.5 to up-regulation or ≤0.66 to down-regulation).

Involved Pathway	Gene Symbol	Treatment (µg/mL)	72 h Mean ± SD	Entrez Gene Name
Metabolism (activation/detoxification)	*CYP1A1*	0.5 CYN 1 MCLR 0.5 CYN + 1 MCLR B(a)P 30 µM	**2.59 ± 0.89** 1.03 ± 0.13 **2.53 ± 1.53** 48.87 ± 0.81 ****	Cytochrome P450 family 1 Subfamily A polypeptide 1
	*UGT1A1*	0.5 CYN 1 MCLR 0.5 CYN + 1 MCLR B(a)P 30 µM	0.96 ± 0.45 0.97 ± 0.25 1.20 ± 0.69 2.94 ± 1.15 *	UDP glucuronosyltransferase 1 family polypeptide A1
Immediate-early response/signaling	*JUNB*	0.5 CYN 1 MCLR 0.5 CYN + 1 MCLR B(a)P 30 µM	1.22 ± 0.26 1.10 ± 0.24 1.16 ± 0.35 2.60 ± 0.45 ***	Jun B proto-oncogene
DNA damage responsive	*TP53*	0.5 CYN 1 MCLR 0.5 CYN + 1 MCLR B(a)P 30 µM	0.76 ± 0.02 **** 0.96 ± 0.02 0.75 ± 0.06 **** 1.16 ± 0.04 **	Tumor protein P53
	*MDM2*	0.5 CYN 1 MCLR 0.5 CYN + 1 MCLR B(a)P 30 µM	1.25 ± 0.29 1.08 ± 0.08 1.11 ± 0.21 1.39 ± 0.08	MDM2 oncogene, E3 ubiquitin-protein ligase
	*CDKN1A*	0.5 CYN 1 MCLR 0.5 CYN + 1 MCLR B(a)P 30 µM	2.44 ± 0.30 *** 1.12 ± 0.24 2.47 ± 0.43 *** 8.61 ± 0.16 ****	Cyclin-Dependent Kinase Inhibitor 1A
	*GADD45A*	0.5 CYN 1 MCLR 0.5 CYN + 1 MCLR B(a)P 30 µM	3.16 ± 0.54 ** 1.11 ± 0.10 3.06 ± 0.89 ** 3.76 ± 0.75 ***	Growth arrest and DNA damage-inducible gene, alpha
